# Intermittent hypoxia–driven gut microbial and metabolic remodeling in obstructive sleep apnea: mechanisms and evidence across target organs

**DOI:** 10.3389/fcimb.2026.1965973

**Published:** 2026-10-26

**Authors:** Mengqi Wu, Lingyong Chen, Jiye Zhou, Hui Huang, Xingyu Chen, Shanhong Wan, Xiaotao Xie, Minhua Zhou, Zunjing Zhang

**Affiliations:** 1 Department of Respiratory and Critical Care Medicine, Lishui Hospital of Traditional Chinese Medicine Affiliated to Zhejiang University of Traditional Chinese Medicine, Lishui, China; 2 The Second School of Clinical Medicine, Zhejiang Chinese Medical University, Hangzhou, China

**Keywords:** gut microbiota, intermittent hypoxia, microbial metabolism, obstructive sleep apnea, target-organ injury

## Abstract

Obstructive sleep apnea (OSA) exposes tissues to recurrent intermittent hypoxia (IH) and is associated with multisystem morbidity. Growing evidence implicates the gut microbiota and microbial metabolism in these consequences, but their contribution to target-organ injury remains uncertain. This narrative review examines how IH remodels the intestinal environment and evaluates the evidence linking these changes to systemic injury, distinguishing preclinical experimental findings from human clinical observations. IH can disturb intestinal oxygen homeostasis, impair epithelial barrier function, and alter microbial and host–microbial metabolic pathways involving short-chain fatty acids, bile acids, and trimethylamine N-oxide. These changes may influence distant tissues through metabolic signaling and immune activation. Preclinical studies support a functional microbial contribution to selected cardiovascular, metabolic, and neurocognitive phenotypes, but mechanistic resolution and applicability vary with exposure conditions, diet, and host background. Human studies remain predominantly observational and do not establish microbial mediation of OSA-related organ injury. Overall, the gut microbiota is best regarded as a context-dependent contributor and amplifier of IH-related injury, rather than a universal mediator. A focus on microbial function and host–microbial co-metabolism may be more informative than inconsistent taxonomic signatures. Longitudinal studies and targeted interventions are needed to establish clinical relevance and determine whether microbiome-directed approaches can complement established OSA treatment.

## 1 Introduction

Obstructive sleep apnea (OSA) is a common sleep-related breathing disorder characterized by recurrent partial or complete collapse of the upper airway during sleep. Globally, an estimated 936 million adults aged 30–69 years have mild-to-severe OSA, including approximately 425 million with moderate-to-severe disease (Benjafield et al., 2019). Recurrent upper-airway obstruction leads to intermittent hypoxia (IH) and reoxygenation, fluctuations in intrathoracic pressure, sleep fragmentation, and sympathetic activation. Through downstream processes including oxidative stress, systemic inflammation, endothelial dysfunction, and metabolic dysregulation, OSA is associated with a broad range of adverse outcomes, including hypertension, coronary artery disease, heart failure, stroke, metabolic abnormalities, and neurocognitive impairment (Gottlieb and Punjabi, 2020; Yeghiazarians et al., 2021; Schipper et al., 2021; Punjabi et al., 2009). Among these pathophysiological perturbations, recurrent hypoxia–reoxygenation represents a major mechanistic link between nocturnal respiratory events and systemic tissue injury (Dewan et al., 2015).

The apnea–hypopnea index (AHI) primarily quantifies the frequency of respiratory events and does not adequately capture event duration, the depth of oxygen desaturation, or the cumulative burden of hypoxemia. Hypoxic burden, which integrates the magnitude and duration of oxygen desaturation, has shown stronger associations with cardiovascular events and mortality than AHI in several large cohort studies (Azarbarzin et al., 2019; Labarca et al., 2023; Watanabe et al., 2017; Hui et al., 2024). These observations suggest that IH and its cumulative burden may more closely reflect the biologically relevant exposure underlying systemic injury in OSA than respiratory-event frequency alone.

In recent years, the gut microbiota has emerged as a potential contributor to the systemic pathophysiology of OSA. Experimental studies have shown that IH can alter gut microbial composition (Moreno-Indias et al., 2015), while models combining intermittent hypoxia and hypercapnia have further demonstrated that microbial alterations occur in parallel with remodeling of bile acids, fatty acids, and other host–microbial co-metabolites (Tripathi et al., 2018). Clinical studies have likewise identified abnormalities in microbial composition, functional potential, intestinal barrier integrity, and inflammatory markers in patients with OSA (Ko et al., 2019; Li et al., 2023b). Large-scale metagenomic studies further suggest that hypoxemia-related parameters, including time spent with oxygen saturation below 90% (T90) and the oxygen desaturation index, are more strongly associated with microbial species and functional pathways than measures based primarily on respiratory-event frequency (Baldanzi et al., 2023; Guo et al., 2024). However, the reproducibility of specific taxonomic signatures across populations remains limited. Although systematic reviews and meta-analyses support overall alterations in gut microbial community structure in OSA, no stable and consistently replicated taxonomic signature has yet been identified (Tahmasebi et al., 2025). Accordingly, microbial function and metabolic output may provide greater mechanistic insight than the abundance of individual taxa alone.

Interventional studies provide further evidence that OSA- or IH-associated alterations in the gut microbiota may contribute to target-organ injury. Dysbiotic microbial communities can amplify hypertension and transfer selected vascular abnormalities to recipient animals (Durgan et al., 2016; Badran et al., 2023). Germ-free conditions can attenuate aortic atherosclerosis induced by OSA-like exposure (Xue et al., 2025a), while chronic intermittent hypoxia (CIH)-associated changes in the gut microbiota and bile acid metabolism may contribute to hepatic steatosis (Zhang et al., 2026). Nevertheless, relatively direct causal evidence remains concentrated in selected cardiovascular and metabolic phenotypes. In this review, we consider IH as a quantifiable ecological selection pressure acting on the intestinal ecosystem and summarize its effects on microbial ecology and metabolic function. We further compare the contribution of the gut microbiota across OSA-related target organs using an evidence framework that considers association, phenotype transferability, microbiota dependence, defined microbial or metabolite validation, and pathway-specific mechanistic evidence.

### 1.1 Literature search and study selection

A structured PubMed search was conducted to identify studies relevant to this narrative review, covering the period from database inception through August 2026. MeSH and title/abstract terms related to obstructive sleep apnea, intermittent hypoxia or hypoxemia, sleep fragmentation, gut microbiota, intestinal barrier dysfunction, short-chain fatty acids, bile acids, trimethylamine/trimethylamine N-oxide, endotoxemia, and selected microbial co-metabolites were combined. No publication-date, language, or species filters were applied; the complete search strategy is provided in **Supplementary Methods S1**.

Records were screened by title and abstract for relevance to intestinal remodeling, microbial metabolism, and target-organ outcomes. Original human and experimental studies were considered when they examined OSA or relevant experimental exposures together with gut microbial, barrier, or metabolic measures and organ-related outcomes. Studies restricted to oral or airway microbiota or unrelated exposures were excluded. Reviews and broader physiological studies were used for background, and preprints were not included in the main evidence assessment. Studies were assigned to organ-specific sections according to the outcomes measured, with multiple organ assignments permitted when appropriate. Human and preclinical evidence and different exposure paradigms, including IH, IHC, mechanical OSA, sleep fragmentation, and dietary co-exposures, were considered separately. Both supportive and null findings were considered, with greater emphasis on transplantation, microbiota depletion, defined microbial or metabolite interventions, and pathway perturbation when interpreting mechanistic evidence. The synthesis was qualitative and narrative; no formal risk-of-bias scoring or quantitative pooling was performed.

## 2 Intestinal remodeling and systemic signaling in OSA: effects of IH and interacting exposures

### 2.1 The intestinal oxygen environment under homeostatic conditions

The healthy colon is characterized by a radial oxygen gradient that progressively decreases from the mucosa toward the intestinal lumen. Oxygen diffuses from the highly vascularized mucosal tissue into the lumen but is continuously consumed by epithelial cells and mucosa-associated microorganisms. As a result, the epithelial surface remains physiologically hypoxic, whereas the bulk luminal environment approaches strict anaerobiosis, thereby creating relatively stable ecological niches for microorganisms with different oxygen tolerances (Albenberg et al., 2014).

Mature colonocytes preferentially utilize microbiota-derived butyrate as an energy source and consume substantial amounts of oxygen through mitochondrial β-oxidation and oxidative phosphorylation, thereby limiting oxygen diffusion into the intestinal lumen (Donohoe et al., 2011; Kelly et al., 2015; Ornelas et al., 2023). The physiological hypoxia generated by butyrate metabolism also contributes to the stabilization of hypoxia-inducible factor (HIF), which promotes tight-junction integrity, mucus production, and tissue repair (Kelly et al., 2015; Karhausen et al., 2004; Robinson et al., 2008; Saeedi et al., 2015). When butyrate production or colonocyte oxidative metabolism is impaired, epithelial oxygen consumption decreases, increasing oxygen availability at the mucosal–luminal interface and favoring facultative anaerobes. In parallel, inflammatory responses can increase host-derived nitrate, providing an additional respiratory electron acceptor for facultative anaerobes, including members of the Enterobacteriaceae (Byndloss et al., 2017; Winter et al., 2013). Thus, the intestinal oxygen landscape is a dynamic property jointly maintained by mucosal perfusion, epithelial metabolism, and microbial fermentation.

### 2.2 Intermittent hypoxia–induced alterations in the gut microbiota

IH can perturb the normally low-oxygen intestinal microenvironment and impose cyclical fluctuations in local oxygen availability. Repeated reductions and recovery of arterial oxygenation can be transmitted to the intestinal wall, altering mucosal oxygen delivery, epithelial metabolism, and oxygen diffusion toward the lumen (Moreno-Indias et al., 2015; Glover et al., 2016; Kelly et al., 2015; Litvak et al., 2018). Moreno-Indias et al. showed that oxygen tension in fecal material close to the mucosa fluctuated in parallel with changes in inspired oxygen concentration, and that prolonged IH exposure was accompanied by marked alterations in microbial diversity and community structure (Moreno-Indias et al., 2015). These findings suggest that IH can act as a direct environmental selection pressure on the local intestinal ecosystem.

However, this selective pressure cannot be reduced to a simple shift from “strict anaerobes” toward “facultative anaerobes.” Microbial responses are shaped by multiple factors, including oxygen tolerance, substrate utilization, bile acid availability, intestinal transit, and host inflammatory status. Multi-omics studies using models of intermittent hypoxia combined with hypercapnia have shown that microbial remodeling occurs in parallel with alterations in bile acids, fatty acids, aromatic compounds, and lipid metabolites, with some features reproduced across different rodent models (Tripathi et al., 2018, 2019). These observations suggest that OSA-like intermittent gas perturbations can remodel broader host–microbial co-metabolic networks rather than producing isolated fluctuations in a limited number of bacterial genera. However, because these models incorporate hypercapnia, the observed effects cannot be attributed to IH alone.

Notably, some microbiota abnormalities and elevated circulating endotoxin levels persist after restoration of normoxia (Moreno-Indias et al., 2016), suggesting that ecological recovery may lag behind normalization of blood oxygenation. Such persistence may reflect ecological niche occupancy, cross-feeding interactions, and sustained host feedback mechanisms.

### 2.3 Intestinal barrier dysfunction and inflammatory responses

Repeated hypoxia–reoxygenation can enhance oxidative stress, induce mitochondrial injury in intestinal epithelial cells, and activate inflammatory signaling pathways such as NF-κB (Wu et al., 2016; Wang et al., 2024). Animal studies have shown that IH can cause villous and microvillous injury, reduce goblet cell numbers, disrupt tight-junction architecture, and increase intestinal permeability (Wu et al., 2016; Li et al., 2023c; Xue et al., 2025b; Xu et al., 1999; Wang et al., 2024). These findings indicate that IH may influence the host indirectly through microbiota dysregulation while also exerting direct damaging effects on the intestinal mucosal barrier.

Following barrier disruption, microbial components such as lipopolysaccharide can more readily enter the portal and systemic circulation, where they may amplify inflammation through TLR4-related pathways. In turn, inflammation-derived nitrate can promote respiratory growth of facultative anaerobes, creating a self-reinforcing interaction among microbial dysbiosis, barrier dysfunction, and inflammation (Byndloss et al., 2017; Winter et al., 2013). Clinical studies have also reported elevated levels of intestinal fatty acid-binding protein (I-FABP), D-lactate, and lipopolysaccharide-binding protein in subsets of patients with OSA, with these markers correlating with OSA severity, nocturnal hypoxemia, or microbial alterations (Barceló et al., 2016; Kheirandish-Gozal et al., 2014; Kong et al., 2018; Li et al., 2023b). However, these biomarkers are also influenced by obesity, metabolic status, diet, and medication use, and most available clinical studies are cross-sectional, leaving the direction of causality unresolved.

Whether these intestinal alterations contribute causally to distal-organ phenotypes varies substantially across organs and requires evaluation using microbiota transplantation and targeted microbiome interventions, as discussed below.

### 2.4 Sleep fragmentation and circadian disruption as modifiers of IH-associated microbiome remodeling

The composition and metabolic activity of the gut microbiota exhibit circadian rhythmicity and are influenced by feeding–fasting cycles, bile acid secretion, intestinal motility, and the host biological clock (Thaiss et al., 2014; Zarrinpar et al., 2014). In models combining intermittent hypoxia with hypercapnia, rhythmic oscillations in the microbiota and in metabolites such as bile acids, glycerophospholipids, and acylcarnitines can become attenuated, phase shifted, or completely lost (Allaband et al., 2021; Tripathi et al., 2018).

The pathophysiology of clinical OSA is not determined by IH alone. Sleep fragmentation itself can increase food intake, induce microbial dysbiosis and intestinal barrier dysfunction, and promote adipose tissue inflammation and insulin resistance. Microbiota associated with sleep fragmentation can also transfer selected metabolic phenotypes to germ-free recipients (Poroyko et al., 2016). Parallel experimental models of CIH and sleep fragmentation further demonstrate that their effects on the microbiota, circulating metabolites, adipose tissue, and liver are not fully overlapping and can be substantially modified by a high-fat diet (Wang et al., 2022). Therefore, interpretation of microbiome alterations in clinical OSA requires careful distinction between the effects of IH and those of sleep disruption, circadian dysregulation, diet, and obesity.

### 2.5 Potential systemic routes linking the gut to distant organs

IH-associated intestinal alterations may influence distant organs through metabolites, microbial components, immune cells, and neuroendocrine signaling. Short-chain fatty acids, trimethylamine–trimethylamine N-oxide co-metabolites, secondary bile acids, and aromatic amino acid derivatives can enter the portal or systemic circulation. Studies in humans and germ-free animals have demonstrated the intestinal absorption and systemic availability of SCFAs, microbiota-dependent generation of trimethylamine N-oxide (TMAO), and microbial regulation of the bile acid pool and circulating aromatic metabolites (Bloemen et al., 2009; Tang et al., 2013; Sayin et al., 2013; Dodd et al., 2017). These findings provide a biological basis for the systemic dissemination of gut-derived signals, although they do not constitute OSA-specific validation.

Barrier disruption may further increase systemic exposure to peptidoglycan, lipopolysaccharide, and bacterial extracellular vesicles, which can influence distal immune responses through pathways including NOD1. Gut-derived extracellular vesicles containing microbial DNA have also been shown to promote adipose tissue inflammation and insulin resistance (Clarke et al., 2010; Tulkens et al., 2020; Luo et al., 2021). In addition, the gut microbiota can modulate the balance between Th17 and regulatory T cells and influence central and stress-related responses through the vagus nerve and the hypothalamic–pituitary–adrenal axis (Ivanov et al., 2009; Atarashi et al., 2011; Bravo et al., 2011; Sudo et al., 2004). The actual contribution of these general mechanisms to IH and clinical OSA, however, remains to be established through microbiota transplantation, cellular tracing, and pathway-specific blockade studies. These intestinal ecological, barrier, and metabolic alterations and their potential routes of systemic dissemination are summarized in **Figure 1**.

**Figure 1:**
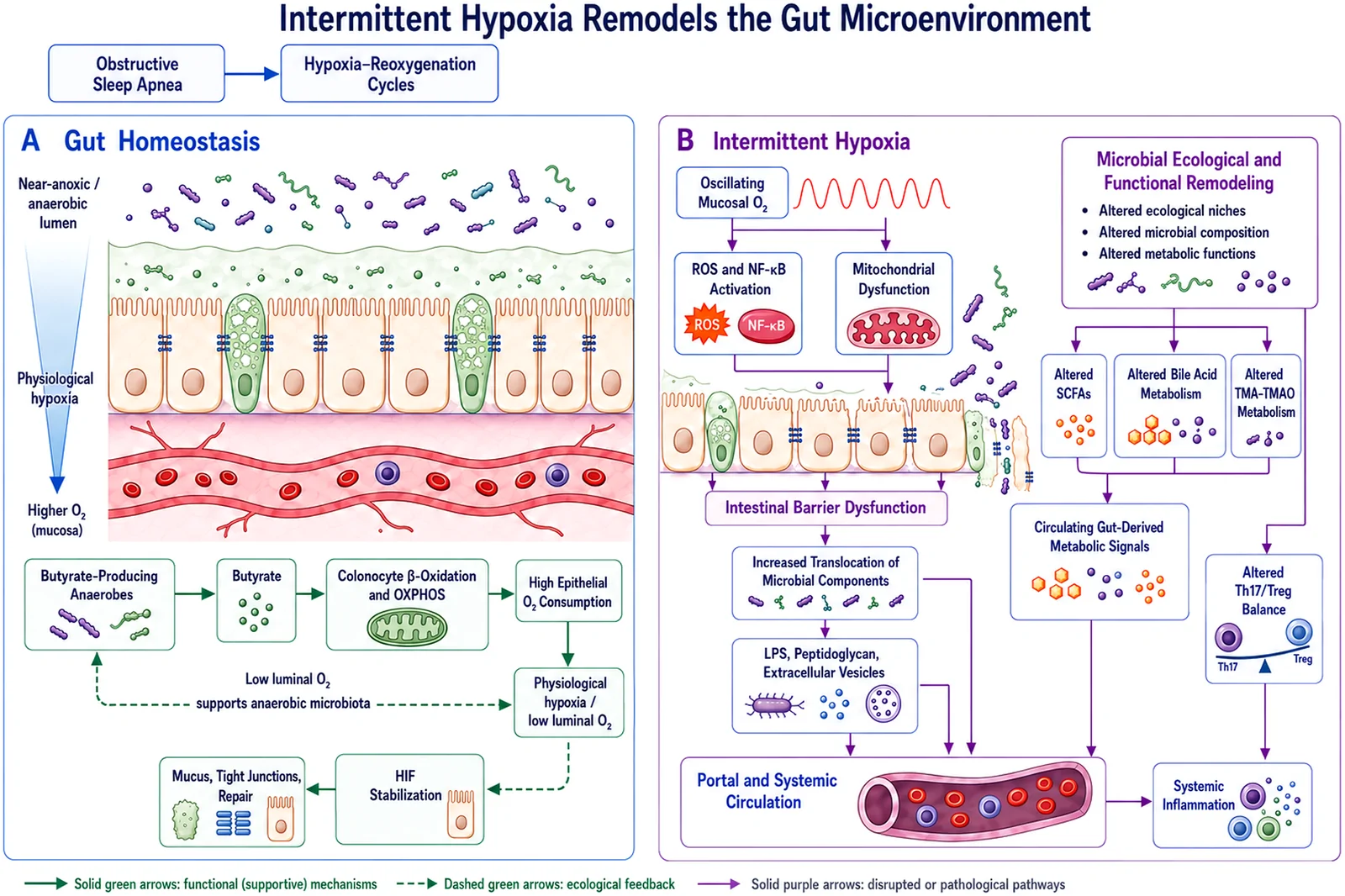
Intermittent hypoxia remodels the intestinal microenvironment and promotes systemic dissemination of gut-derived signals. **(A)** Under homeostatic conditions, microbiota-derived butyrate supports colonocyte oxidative metabolism, maintains low luminal oxygen availability, and promotes HIF-dependent epithelial barrier integrity. **(B)** Intermittent hypoxia–reoxygenation perturbs intestinal oxygen homeostasis, promotes oxidative and inflammatory stress, impairs barrier function, and is associated with microbial and metabolic remodeling involving SCFAs, bile acids, and TMA–TMAO pathways. These changes may facilitate systemic exposure to microbial products, metabolites, extracellular vesicles, and immune signals. Green arrows indicate supportive physiological mechanisms, dashed green arrows ecological feedback, and purple arrows disrupted or pathological pathways.

### 2.6 Functional and mechanistic links between microbial metabolism and target-organ injury

Microbial metabolites can connect intestinal remodeling to target-organ dysfunction through epithelial, metabolic, and immune signaling. Short-chain fatty acids (SCFAs) illustrate these interconnected effects. Butyrate supports epithelial oxygen consumption and hypoxia-inducible factor-dependent barrier protection, while also promoting colonic regulatory T-cell differentiation through epigenetic regulation at the Foxp3 locus (Kelly et al., 2015; Furusawa et al., 2013). Reduced butyrate availability could therefore compromise both epithelial defense and immune tolerance, although these mechanisms were established outside OSA models. More directly relevant to OSA, cecal acetate supplementation prevented intestinal inflammation and hypertension in high-fat-diet-fed rats subjected to mechanically induced OSA (Ganesh et al., 2018). This supports a protective effect of acetate in that setting, without establishing that different SCFAs act interchangeably or that their depletion universally mediates OSA-related injury.

Bile acids provide another link between microbial activity and host metabolic regulation. Microbial modification of the host-derived bile acid pool changes its receptor-signaling properties. In mice, microbial regulation of tauro-β-muricholic acid, an FXR antagonist, influences intestinal FXR–FGF15 signaling and hepatic bile acid synthesis (Sayin et al., 2013). Separately, TGR5 activation promotes intestinal GLP-1 secretion and improves glucose tolerance in experimental models (Thomas et al., 2009). These mechanisms make bile acid remodeling relevant to hepatic and systemic metabolic dysfunction, but their involvement in OSA requires specific validation. In lean mice, prolonged CIH induced hepatic steatosis that was attenuated by antibiotics, alongside coordinated changes in Bacteroides uniformis, deoxycholic acid, and hepatic PPAR-related pathways (Zhang et al., 2026). These findings implicate microbiota–host metabolic interactions but do not establish a causal sequence linking this bacterial species, a particular bile acid, and receptor-dependent liver injury.

The TMA–TMAO pathway likewise depends on sequential microbial and host metabolism. Microorganisms convert dietary precursors, including choline-containing nutrients, into trimethylamine (TMA), which hepatic flavin-containing monooxygenases, particularly FMO3, oxidize to trimethylamine N-oxide (TMAO) (Badran et al., 2023). Experimental evidence outside OSA shows that TMAO enhances agonist-induced platelet calcium mobilization and platelet responsiveness, providing a mechanism for increased thrombosis potential (Zhu et al., 2016). In an IH-related microbiota transplantation study, recipient mice developed elevated blood pressure and vascular dysfunction accompanied by increased TMAO (Badran et al., 2023). However, transferability of the cardiovascular phenotype does not identify TMAO as its mediator. Selective inhibition of microbial TMA production combined with metabolite rescue would help determine whether this pathway contributes causally to IH-associated vascular abnormalities.

Intestinal barrier dysfunction may amplify these metabolic effects by increasing host exposure to microbial inflammatory signals. In mice receiving microbiota from OSA donor groups, hypertension and aortic injury accompanied reduced intestinal tight-junction protein expression and increased LPS-related and aortic TLR4/NF-κB signaling markers (Zhang et al., 2025). This pattern supports a potential connection between barrier impairment and vascular inflammation, but does not establish that LPS translocation or TLR4 activation is necessary for the transferred phenotype. Human associations between OSA, microbial alterations, and circulating intestinal injury or permeability-related markers provide clinical context, while remaining insufficient to demonstrate microbial translocation or causal mediation of organ injury (Li et al., 2023b).

Immune regulation offers an additional route from intestinal disturbances to distant-organ dysfunction. In a mechanically induced OSA rat model, altered Treg and Th17 responses accompanied intestinal and brain inflammation; tracking experiments supported trafficking of gut-derived T cells, and IL-17A neutralization prevented hypertension and attenuated inflammation (Ayyaswamy et al., 2023). This establishes a functional contribution of IL-17A-dependent inflammation in that model, although the initiating microbial signals remain unresolved. In a separate ketogenic-diet–hypoxia model, microbiota transplantation and Bilophila wadsworthia colonization supported a microbial contribution to cognitive impairment, while inhibition of Th1-cell development prevented the associated deficits (Olson et al., 2021). Thus, immune mechanisms can connect microbial changes to organ phenotypes, but the responsible pathways depend on dietary and experimental conditions.

These processes should be interpreted within a host–microbial co-metabolic framework: metabolite exposure depends on microbial production together with host absorption, transformation, and tissue utilization. Human measurements across the gut and liver demonstrated hepatic uptake of propionate and butyrate, showing why intestinal availability cannot be equated with systemic exposure (Bloemen et al., 2009). Fecal metabolite concentrations therefore cannot independently establish production rates or target-organ exposure, and microbial abundance does not establish metabolic flux. Paired intestinal and circulating measurements, isotope tracing, and selective microbial or host-pathway interventions would strengthen mechanistic attribution. Overall, current evidence supports several interacting routes of microbiota-associated injury, with the strongest causal support arising from selected preclinical models rather than human OSA studies.

## 3 Mechanistic links between the gut microbiota and OSA-related target-organ injury

The concurrent occurrence of gut microbial alterations and target-organ injury does not establish causality, because IH can also directly damage organs through tissue hypoxia, oxidative stress, sympathetic activation, and hemodynamic disturbances. The following sections therefore focus on evidence from microbiota transplantation, microbiota depletion, germ-free models, single-strain colonization, and metabolite interventions to evaluate the functional contribution of the gut microbiota to injury across different OSA target organs. Because most of these functional and mechanistic approaches have been performed in animal models, evidence derived from them is referred to here as preclinical experimental evidence, whereas human clinical findings are identified separately.

### 3.1 Cardiovascular injury: hypertension, vascular dysfunction, atherosclerosis, and myocardial effects

Hypertension and vascular dysfunction are among the cardiovascular phenotypes for which microbiota-related evidence is currently strongest. Durgan et al. showed that, in a mechanical OSA model, OSA exposure under a standard diet did not induce sustained hypertension, whereas the combination of OSA and a high-fat diet led to gut dysbiosis, depletion of short-chain fatty acid (SCFA)-producing bacteria, and elevated blood pressure. Broad-spectrum antibiotic treatment prevented the development of hypertension, while transfer of cecal microbiota from OSA-exposed animals to standard-diet recipients that remained exposed to OSA reproduced the hypertensive response (Durgan et al., 2016). These findings therefore primarily support a role for the altered microbiota in amplifying the pressor effects of OSA rather than independently inducing hypertension in the absence of OSA exposure.

SCFA deficiency may represent an important component of this pathway. In high-fat-diet-fed rats subjected to mechanically induced OSA, supplementation with Clostridium butyricum or the resistant starch Hylon VII prevented blood-pressure elevation. Direct cecal acetate supplementation also prevented intestinal inflammation and hypertension, supporting a protective effect of acetate in this experimental setting (Ganesh et al., 2018). SCFAs can also regulate blood pressure through GPR41 and Olfr78, although the effects mediated by these receptors may differ in direction (Pluznick et al., 2013; Pluznick, 2014). Accordingly, the protective effects of acetate are likely to involve multiple mechanisms, including modulation of barrier integrity, immunity, vascular function, and autonomic regulation, rather than a single receptor-dependent pathway.

Microbiota transplantation studies using isolated IH exposure provide further evidence for a functional vascular contribution. Microbiota from IH-exposed donors transferred elevated blood pressure and impaired aortic and coronary vasorelaxation to recipients that had not themselves been exposed to hypoxia, accompanied by reduced acetate and increased trimethylamine N-oxide (TMAO). The probiotic mixture VSL#3 attenuated abnormalities in microbiota recipients but provided limited protection in animals directly exposed to IH (Badran et al., 2023). These findings support a contribution of the gut microbiota to IH-related vascular dysfunction, while also underscoring that microbial effects do not replace the direct actions of hypoxia on the endothelium, sympathetic nervous system, and redox homeostasis.

TMAO, lipopolysaccharide (LPS), and immune cells have been proposed as candidate mediators. The microbiota-dependent capacity for TMAO generation can be transferred by microbiota transplantation and has been linked to enhanced platelet reactivity and thrombosis (Tang et al., 2013; Zhu et al., 2016). However, OSA/IH-specific studies using inhibition of microbial trimethylamine production or downstream pathway blockade remain lacking; TMAO therefore cannot yet be regarded as an OSA-specific microbiota-derived mediator. Experimental studies also suggest that OSA-associated microbiota may promote hypertension and aortic injury through the LPS–TLR4/NF-κB pathway (Zhang et al., 2025). In mechanical OSA models, reduced intestinal regulatory T cells (Tregs), enhanced Th17/IL-17A responses, and migration of gut-derived helper T cells have also been observed, while IL-17A neutralization attenuated blood pressure elevation and inflammatory abnormalities (Ayyaswamy et al., 2023). These pathways require further validation using tissue-specific genetic models, LPS tracing, and immune-cell lineage tracking.

Evidence for atherosclerosis derives mainly from combined experimental models. In ApoE-deficient mice exposed to a high-fat, high-cholesterol diet together with intermittent hypoxia and hypercapnia (IHC), germ-free conditions markedly attenuated aortic atherosclerosis and were accompanied by changes in Akkermansiaceae, Muribaculaceae, and secondary bile acids (Xue et al., 2025a). The protective effect was more pronounced in the aorta than in the pulmonary artery, while intermittent hypoxia and intermittent hypercapnia exerted distinct effects on different vascular beds and microbial metabolic profiles (Xue et al., 2021). These findings indicate that the effects observed in IHC models cannot be attributed entirely to IH. Moreover, direct transfer of the atherosclerotic phenotype by microbiota transplantation and bidirectional manipulation of candidate bile acid pathways remain to be demonstrated.

Overall, OSA-related hypertension and vascular dysfunction are supported by preclinical evidence from microbiota transplantation, antibiotics, probiotics, prebiotics, acetate supplementation, and immune intervention, whereas atherosclerosis remains more dependent on dietary and IHC-related experimental contexts. Although conventionally colonized and germ-free mice exhibit distinct left ventricular transcriptional responses following IHC exposure, there is currently no evidence that the microbiota can transfer or reverse structural or functional myocardial injury (Zhou et al., 2021). Microbiota-related myocardial injury therefore remains at an exploratory mechanistic stage.

### 3.2 Metabolic and hepatic injury: from insulin resistance to gut–liver metabolic remodeling

IH can directly impair insulin signaling through adipose tissue hypoxia, oxidative stress, and immune-cell infiltration, while the gut microbiota may further amplify these metabolic abnormalities. Even in the absence of substantial increases in adiposity, IH can promote macrophage infiltration into visceral adipose tissue, increase reactive oxygen species production, and induce insulin resistance, with only partial reversal after restoration of normoxia (Carreras et al., 2015; Gileles-Hillel et al., 2017). The gut microbiota should therefore be considered a contributing factor rather than the sole mechanism.

Khalyfa et al. showed that IH remodeled the fecal microbiota and metabolome, increased colonic permeability, and altered the cargo of circulating extracellular vesicles. Transfer of microbiota from IH-exposed donors to normoxic recipients induced barrier abnormalities and insulin resistance, while plasma extracellular vesicles from IH-exposed animals suppressed insulin-stimulated AKT phosphorylation in adipocytes; physical activity partially ameliorated these alterations (Khalyfa et al., 2021). Clinically, plasma extracellular vesicles obtained from patients with sleep-disordered breathing were also shown to inhibit AKT phosphorylation in human adipocytes (Khalyfa et al., 2018). However, the intestinal origin of these vesicles and their direct relationship to microbial alterations have not been established. They should therefore currently be regarded as candidate carriers rather than confirmed gut-derived mediators, and the term “extracellular vesicles” is more appropriate than “exosomes” in the absence of clear evidence regarding biogenesis (Welsh et al., 2024).

Sleep fragmentation can independently increase food intake, disrupt the intestinal barrier, promote systemic and adipose tissue inflammation, and induce insulin resistance. Microbiota associated with sleep fragmentation can also transfer selected metabolic phenotypes to germ-free recipients (Poroyko et al., 2016). When CIH and sleep fragmentation are modeled separately, both alter the microbiota, circulating metabolome, adipose tissue, and liver, but the affected tryptophan, fatty acid, branched-chain amino acid, and bile acid pathways are not identical and are substantially modified by a high-fat diet (Wang et al., 2022). The gut–adipose axis in clinical OSA is therefore likely to reflect the combined influence of IH, sleep disruption, feeding behavior, and obesity.

The liver is directly exposed to gut-derived signals through the portal circulation, but CIH can also injure hepatocytes independently of the gut. In non-obese animals, CIH alone can induce oxidative stress, glycogen accumulation, and mild hepatic injury, and can exacerbate inflammation and lipid deposition in the presence of a high-fat diet or pre-existing steatosis (Savransky et al., 2007b, 2007; Drager et al., 2011). Remodeling of fatty acid, amino acid, and energy metabolism, together with RIPK3-dependent programmed necrosis and imbalance of Nrf2/NF-κB signaling, has also been implicated in IH-related fatty liver injury (Chen et al., 2021; Zhang et al., 2021). Thus, microbiota-related mechanisms must be distinguished from the direct hypoxic effects of CIH on hepatocytes.

Multi-omics studies using IHC and CIH models have shown that microbial alterations frequently occur in parallel with changes in bile acids, fatty acids, and other lipid metabolites, with some features reproduced across different rodent models (Tripathi et al., 2018, 2019). The hepatic and circulating metabolic effects of CIH and sleep fragmentation are likewise jointly shaped by exposure type and dietary background (Wang et al., 2022). Such observations are useful for identifying candidate pathways but cannot substitute for microbiota transplantation or targeted mechanistic interventions.

A recent study in lean mice maintained on standard chow showed that 8 weeks of CIH predominantly induced hepatic metabolic and transcriptional changes, whereas 16 weeks resulted in abnormalities in glucose metabolism, hepatic lipid accumulation, and inflammation. Broad-spectrum antibiotic treatment partially attenuated hepatic steatosis and inflammation. CIH was associated with depletion of Bacteroides uniformis and elevation of deoxycholic acid (DCA), both of which showed phenotype-aligned, antibiotic-sensitive changes, together with alterations in peroxisome proliferator-activated receptor (PPAR)-related pathways (Zhang et al., 2026), suggesting that prolonged CIH may induce coordinated remodeling of the microbiota–bile acid–liver axis. However, the study did not demonstrate that loss of B. uniformis directly caused DCA accumulation, nor did it establish DCA as a necessary mediator. Antibiotics may also exert direct effects on host immunity, mitochondrial function, and bile acid metabolism. Further studies should test phenotype transferability through transplantation of CIH-associated microbiota and assess specific microbial contributions using defined communities or single-strain interventions. Controlled DCA administration and interventions that selectively reduce DCA formation or availability, followed by DCA rescue, would help distinguish its sufficiency from its necessity. Candidate host pathways, including farnesoid X receptor (FXR), G protein-coupled bile acid receptor 1 (TGR5), and PPAR-related signaling, should be examined using gain- or loss-of-function approaches guided by the observed signaling changes, because the available study did not establish a specific bile acid receptor-mediated mechanism (Zhang et al., 2026).

Overall, a microbiota-related contribution to IH-induced insulin resistance is supported by preclinical microbiota-transplantation evidence, whereas parallel studies of circulating extracellular vesicles identify candidate systemic carriers without yet establishing a gut or microbial origin. Evidence for hepatic steatosis has progressed from multi-omics associations to preclinical antibiotic intervention, but microbiota transfer, single-strain rescue, and bile acid pathway blockade remain lacking. The gut microbiota may therefore contribute to and amplify CIH-related liver injury, but it cannot yet be regarded as the primary driver.

### 3.3 Neurocognitive injury: gut–brain immune and metabolic signaling

IH can directly impair cognition through cerebral hypoxia, oxidative stress, cerebrovascular dysfunction, and neuroinflammation, while the gut microbiota may modulate these processes through immune cells, circulating inflammatory signals, and microbial metabolites. Basic studies have shown that the gut microbiota can influence blood–brain barrier integrity, microglial homeostasis, and hippocampal neurogenesis (Braniste et al., 2014; Erny et al., 2015; Möhle et al., 2016).

Transfer of microbiota from CIH-exposed mice to normoxic recipients altered sleep duration and architecture (Badran et al., 2020), suggesting that IH-associated microbial changes can influence central nervous system function, although sleep phenotypes should not be equated with cognitive injury. More direct evidence comes from a model combining IH with a ketogenic diet, in which the exposure promoted enrichment of Bilophila wadsworthia and aggravated learning and memory impairment. Broad-spectrum microbiota depletion attenuated the injury, whereas transfer of the altered microbiota or mono-colonization with B. wadsworthia induced impaired hippocampal long-term potentiation, reduced neurogenesis, and cognitive decline (Olson et al., 2021). Inhibition of T-bet-dependent Th1-cell development prevented these phenotypes, supporting a potential strain–intestinal immune–hippocampal functional axis. However, the interpretation of these findings is constrained by the ketogenic-diet background.

Studies using CIH alone have implicated loss of Bifidobacterium pseudolongum in cognitive impairment. Supplementation with this bacterium restored circulating acetate, reduced microglial activation, suppressed cGAS–STING signaling and neuronal PANoptosis in the hippocampus, and improved cognitive performance; direct acetate supplementation produced similar effects (Sun et al., 2026). Another IH study showed that triacetin attenuated neuroinflammation and cognitive dysfunction through suppression of SETDB1/H3K9me3 as well as NF-κB and STAT3 signaling (Zhao et al., 2025). These findings support a neuroprotective role for acetate but require further validation through bacterial loss-of-function approaches, receptor blockade, and independent replication.

Microbiota derived from animals exposed to sustained hypoxia can increase intestinal and blood–brain barrier permeability and transfer selected memory deficits (Li et al., 2025). Dysbiosis associated with sleep deprivation has also been linked to excessive microglial engulfment of synapses and cognitive decline, while supplementation with Akkermansia muciniphila partially ameliorated these abnormalities (Li et al., 2023a). However, these models differ fundamentally from the cyclical hypoxia–reoxygenation pattern of OSA and should therefore be considered supportive rather than OSA-specific mechanistic evidence.

Overall, microbiota depletion, microbiota transplantation, mono-colonization, and acetate supplementation collectively support a role for the gut microbiota in IH-related neurocognitive abnormalities. The B. wadsworthia–Th1 axis and the B. pseudolongum–acetate–cGAS/STING pathway represent two particularly specific candidate mechanisms. Nevertheless, the available evidence remains limited by specialized dietary conditions, reliance on individual animal models, and insufficient clinical validation. The gut microbiota is therefore more appropriately viewed as a modulator and amplifier of IH-related neurocognitive effects rather than as an alternative to direct cerebral hypoxia, cerebrovascular dysfunction, or sleep fragmentation.

### 3.4 Renal and other target-organ injury: potential links and evidence gaps

The kidney is particularly sensitive to alterations in oxygen delivery and hemodynamics. Clinical studies suggest that nocturnal hypoxemia is associated with accelerated decline in renal function, whereas experimental studies have shown that IH can induce glomerular hypertrophy, tubular injury, and apoptosis, accompanied by altered expression of growth factors including TGF-β1, CTGF, and VEGF (Ahmed et al., 2011; Abuyassin et al., 2018). Ang-(1–7) treatment, TLR4 deficiency, and inhibition of the NLRP3 inflammasome can attenuate CIH-related renal inflammation, oxidative stress, and fibrosis, whereas BNIP3-mediated mitophagy may exert protective effects (Lu et al., 2017; Zhang et al., 2019; Wu et al., 2018). These findings indicate that tissue hypoxia, oxidative stress, inflammation, and fibrotic signaling can themselves account for at least part of IH-related renal injury, although they do not exclude a modulatory contribution from the gut microbiota.

Studies in chronic kidney disease provide a broader mechanistic basis for a gut–kidney connection. Patients with chronic kidney disease exhibit alterations in microbial taxa and functional genes involved in aromatic amino acid metabolism, together with accumulation of gut-derived metabolites such as indoxyl sulfate, p-cresyl sulfate, and TMAO (Wu et al., 2020; Lin et al., 2022; Tang et al., 2015). Experimental studies further suggest that butyrate may suppress renal inflammation and fibrosis, whereas TMAO may promote tubular injury (Tian et al., 2023; Ge et al., 2024). However, these findings derive primarily from non-OSA renal disease models, and circulating metabolite concentrations are themselves strongly influenced by renal clearance. They therefore cannot be directly extrapolated to OSA-related kidney injury. The frequent coexistence of hypertension, obesity, and diabetes in OSA further increases the potential for reverse causation and residual confounding.

To date, there is little evidence that transplantation of microbiota from OSA- or IH-exposed donors into unexposed recipients can reproduce a renal injury phenotype, and evidence from germ-free models, defined microbial communities, single-strain colonization, or microbiota-specific metabolite blockade is also lacking. The gut microbiota should therefore currently be regarded as a candidate modulator of OSA-related kidney injury rather than an established mediator.

Microbiota-related evidence in other organs is similarly limited. For example, adrenal injury in CIH-exposed mice can occur in parallel with changes in microbial composition and predicted metabolic function (Chen et al., 2026), but causality has not been established through microbiota transfer or intervention. For the lung, skeletal muscle, reproductive system, and other target organs, available evidence likewise predominantly supports direct tissue injury from IH, whereas the substantive contribution of the gut microbiota remains to be determined.

### 3.5 Cross-organ comparison of evidence and causal boundaries

For the cross-organ comparison, evidence was classified qualitatively as associative/limited, functional, convergent functional, or mechanistically supported. Associative/limited evidence reflects parallel microbial or metabolic changes without direct microbiota-targeted manipulation; functional evidence requires phenotype transfer or attenuation/reversal following microbiota-directed intervention; convergent functional evidence requires more than one complementary functional approach; and mechanistically supported evidence additionally requires defined microbial or metabolite involvement with pathway-specific validation. Microbiota transplantation alone was therefore considered functional evidence.

Experimental paradigms, including isolated IH/CIH, IHC, mechanical OSA, dietary co-exposures, and recipient exposure status, were considered when interpreting evidence strength and generalizability. Context-dependent and model-limited were used as qualifiers rather than separate evidence levels.

The strength of evidence supporting a role for the gut microbiota in IH-related injury differs substantially across target organs. Hypertension is supported by convergent preclinical functional evidence, whereas vascular dysfunction is supported by preclinical functional evidence: altered microbiota can amplify the hypertensive response during ongoing OSA exposure, transfer selected blood pressure and vasorelaxation abnormalities from IH-exposed donors to unexposed recipients, and be partially modified by antibiotics, probiotics, prebiotics, or acetate supplementation (Durgan et al., 2016; Ganesh et al., 2018; Badran et al., 2023). Insulin resistance is similarly supported by preclinical functional evidence from microbiota transplantation, whereas parallel studies of circulating extracellular vesicles identify candidate systemic carriers but do not yet establish a gut or microbial origin (Khalyfa et al., 2021). These findings support a functional microbiota contribution to selected cardiovascular and metabolic phenotypes in preclinical models, while the available human evidence remains supportive rather than causal.

Evidence for neurocognitive injury extends further to single-strain colonization and metabolite rescue. Bilophila wadsworthia can aggravate cognitive dysfunction through Th1-mediated immune responses in the context of IH combined with a ketogenic diet, whereas supplementation with Bifidobacterium pseudolongum or acetate can improve CIH-associated neuroinflammation and cognitive phenotypes (Olson et al., 2021; Sun et al., 2026; Zhao et al., 2025). However, these findings remain dependent on specialized dietary conditions or individual animal models and lack clinical replication and tissue-specific pathway blockade. The gut–brain axis therefore has mechanistically supported preclinical evidence, although it remains model-limited and its generalizability remains uncertain.

By contrast, preclinical functional evidence for atherosclerosis and hepatic steatosis remains context/model-limited and mechanistically incomplete. Germ-free status attenuates aortic atherosclerosis induced by a high-fat, high-cholesterol diet combined with IHC (Xue et al., 2025a), while broad-spectrum antibiotics partially alleviate hepatic steatosis and inflammation during prolonged CIH exposure (Zhang et al., 2026). However, the former model is confounded by both diet and hypercapnia, whereas the latter has not yet been validated by microbiota transplantation, single-strain rescue, or bile acid pathway blockade. Myocardial, renal, adrenal, and other organ phenotypes remain supported mainly by associative/limited microbiota-specific evidence, with little evidence for phenotype transfer or reversal through microbiota-directed intervention (Zhou et al., 2021; Abuyassin et al., 2018; Chen et al., 2026).

These cross-organ differences indicate that the gut microbiota is unlikely to represent a uniform and indispensable downstream mediator of IH-induced target-organ injury. IH itself can directly injure tissues through hypoxia, oxidative stress, sympathetic activation, hemodynamic disturbance, and cell-death pathways, whereas the contribution of the microbiota is likely to vary according to exposure pattern and duration, diet, obesity, sleep fragmentation, and organ-specific susceptibility. At present, the most plausible interpretation is that the microbiota acts as a context-dependent modulator and amplifier whose contribution depends on the mode of exposure, host metabolic background, and target-organ vulnerability, rather than replacing the direct tissue effects of IH.

These categories are qualitative rather than a formal numerical grading system; concordance across phenotype transferability, microbiota dependence, defined microbial or metabolite manipulation, and pathway-specific validation progressively strengthens causal attribution.

A cross-organ comparison of microbiota-related evidence, including evidence source, experimental context, and evidence appraisal, is summarized in **Table 1**. The relative strength of organ-specific evidence and the distinction between direct IH-induced injury and gut-mediated amplification are illustrated in **Figure 2**.

**Table 1:** Cross-organ comparison of causal and functional evidence linking the gut microbiota to OSA/IH-associated target-organ injury.

Target organ/phenotype	Evidence source/experimental context	Microbiota-related evidence	Phenotype transfer/reversal	Candidate microbial or metabolic pathways	Overall evidence appraisal	Key limitations	Representative refs.
Hypertension	Preclinical: mechanical OSA; high-fat-diet co-exposure; microbiota-transfer recipients remained exposed to OSA	Antibiotic treatment prevented hypertension; cecal microbiota transfer reproduced the hypertensive response during ongoing OSA exposure; *Clostridium butyricum*, resistant starch, and acetate were protective.	Yes. In the mechanical OSA model, transfer reproduced hypertension during continued OSA exposure.	SCFA depletion; acetate; LPS-TLR4/NF-κB; Th17/IL-17A-Treg imbalance	Convergent preclinical functional evidence; context-dependent	High-fat-diet dependence in key experiments; in the principal transfer model, recipients remained exposed to OSA, supporting microbiota-dependent amplification rather than demonstrating that the transferred microbiota alone was sufficient to induce hypertension.	(Durgan et al., 2016; Ganesh et al., 2018; Ayyaswamy et al., 2023; Zhang et al., 2025)
Vascular dysfunction	Preclinical: isolated IH; microbiota transferred to normoxic recipients without subsequent IH exposure	Microbiota from IH-exposed donors transferred elevated blood pressure and impaired aortic and coronary vasorelaxation to normoxic recipients; probiotic intervention partially attenuated recipient abnormalities.	Yes	Reduced acetate; increased TMAO; endothelial and inflammatory signaling	Preclinical functional evidence	Phenotype transfer occurred in normoxic recipients, supporting a microbiota-dependent effect independent of continued IH exposure; however, candidate metabolites have not been validated by pathway-specific blockade or rescue in OSA/IH models.	(Badran et al., 2023)
Atherosclerosis	Preclinical: high-fat/high-cholesterol diet + IHC	Germ-free status markedly attenuated aortic atherosclerosis and altered microbial and bile acid profiles.	Not yet demonstrated by microbiota transfer	Secondary bile acids; microbiome-bile acid signaling	Preclinical functional evidence; context/model-limited	Combined effects of diet and hypercapnia; limited transfer/rescue evidence; findings cannot be attributed to IH alone.	(Xue et al., 2021, 2025)
Myocardial effects	Preclinical: IHC	Germ-free and conventionally colonized mice showed different left ventricular transcriptional responses.	No demonstrated transfer or reversal of myocardial injury	Unresolved	Preclinical associative/limited evidence	Evidence is largely transcriptional; structural and functional myocardial phenotypes have not been microbiota-dependently reproduced.	(Zhou et al., 2021)
Insulin resistance	Preclinical: IH with microbiota transfer to normoxic recipients; Human: sleep-disordered breathing extracellular-vesicle study	Microbiota transfer from IH-exposed donors induced barrier abnormalities and insulin resistance in normoxic recipients; circulating extracellular vesicles impaired insulin-stimulated AKT phosphorylation.	Yes for microbiota transfer	Barrier dysfunction; systemic inflammatory/metabolic signals; extracellular vesicles as candidate carriers	Preclinical functional evidence; human evidence supportive/associative	Human extracellular-vesicle findings are supportive but do not establish a gut or microbial origin; direct IH effects on adipose tissue remain important.	Preclinical IH study: (Khalyfa et al., 2021). Human-derived extracellular vesicles: (Khalyfa et al., 2018).
Hepatic steatosis/inflammation	Preclinical: prolonged CIH on standard chow; supporting IHC/CIH multi-omics models	Antibiotics partially attenuated hepatic steatosis and inflammation; CIH-associated changes involved *Bacteroides uniformis*, DCA, and PPAR-related pathways.	No microbiota transfer demonstrated	*B. uniformis*; deoxycholic acid; bile acid metabolism; PPAR-related signaling	Preclinical functional evidence; context/model-limited	Antibiotics have microbiota-independent effects; no single-strain rescue, phenotype transfer, or pathway-specific validation.	Primary study: (Zhang et al., 2026). Supporting multi-omics/context: (Tripathi et al., 2018, 2019; Wang et al., 2022).
Neurocognitive impairment	Preclinical: CIH; IH + ketogenic-diet co-exposure	Microbiota depletion, transplantation, mono-colonization, bacterial supplementation, and acetate supplementation altered cognitive phenotypes.	Yes	*Bilophila wadsworthia*-Th1 axis; *Bifidobacterium pseudolongum*-acetate-cGAS/STING; microglial activation; PANoptosis	Preclinical mechanistically supported evidence; model-limited	Some findings depend on ketogenic diet or individual animal models; clinical replication and tissue-specific pathway validation remain limited.	(Olson et al., 2021; Zhao et al., 2025; Sun et al., 2026)
Renal injury	Human: nocturnal hypoxemia associations; Preclinical: CIH/IH; non-OSA CKD studies used for mechanistic context	Direct renal injury and inflammatory/fibrotic pathways are well described in OSA/IH models, whereas gut-kidney mechanisms are inferred largely from CKD literature.	Not demonstrated	Possible SCFAs, TMAO, indoxyl sulfate, p-cresyl sulfate	Human associative evidence; microbiota-specific preclinical evidence limited	No OSA/IH-specific microbiota transfer, germ-free validation, defined strain intervention, or microbiota-specific metabolite blockade.	OSA/IH renal injury: (Ahmed et al., 2011; Lu et al., 2017; Abuyassin et al., 2018; Wu et al., 2018; Zhang et al., 2019). Non-OSA gut-kidney context: (Tang et al., 2015; Wu et al., 2020; Lin et al., 2022; Tian et al., 2023; Ge et al., 2024).
Adrenal/other organs	Preclinical: CIH (adrenal); other organs with limited microbiota-specific evidence	Microbial alterations may occur in parallel with adrenal and other tissue injury.	Not demonstrated	Largely undefined	Preclinical associative/insufficient evidence	Causality has not been established through microbiota transfer or targeted intervention.	Adrenal injury: (Chen et al., 2026).

Evidence appraisal was qualitative. Associative/limited evidence indicates parallel microbial or metabolic changes without direct microbiota-targeted manipulation; functional evidence indicates phenotype transfer or attenuation/reversal following microbiota-directed intervention; convergent functional evidence requires support from more than one complementary functional approach; and mechanistically supported evidence additionally requires defined microbial or metabolite involvement with pathway-specific validation. Functional and mechanistic evidence is derived predominantly from preclinical studies, whereas observational human findings are interpreted as associative or supportive. Context-dependent and model-limited are used as qualifiers reflecting differences in exposure paradigm, dietary background, and continued OSA/IH exposure in microbiota recipients rather than as separate evidence levels. Abbreviations: CIH, chronic intermittent hypoxia; DCA, deoxycholic acid; FMT, fecal microbiota transplantation; IH, intermittent hypoxia; IHC, intermittent hypoxia and hypercapnia; LPS, lipopolysaccharide; OSA, obstructive sleep apnea; PPAR, peroxisome proliferator-activated receptor; SCFA, short-chain fatty acid; TMAO, trimethylamine N-oxide.

**Figure 2:**
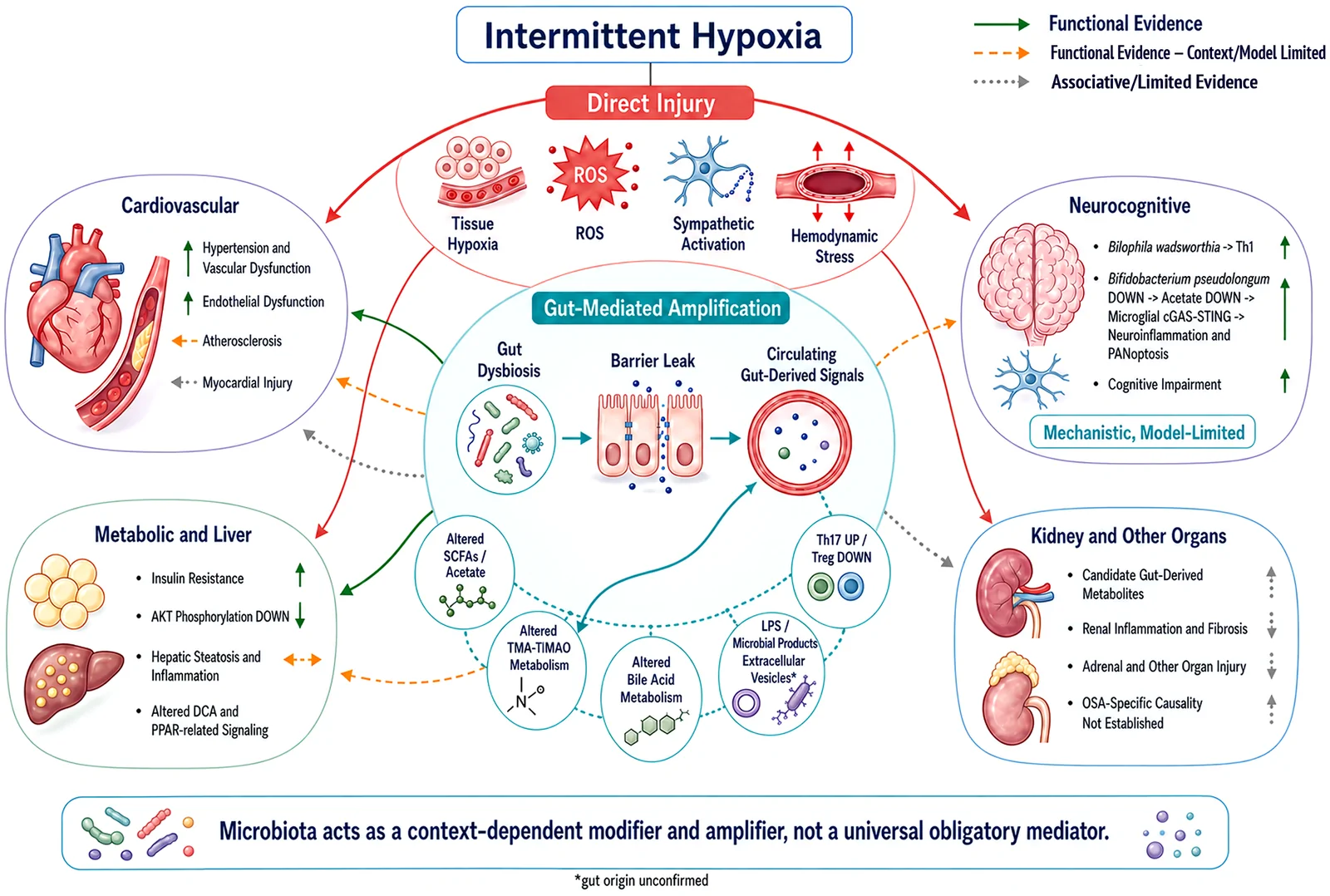
Cross-organ comparison of direct intermittent hypoxia-induced injury and gut microbiota-mediated amplification in OSA. IH can directly injure target organs through tissue hypoxia, oxidative stress, sympathetic activation, and hemodynamic stress, while gut dysbiosis, barrier dysfunction, and altered microbial or host–microbial signals may further amplify systemic injury. Preclinical microbiota-related evidence is most developed for hypertension, vascular dysfunction, and insulin resistance; neurocognitive evidence is mechanistically supported but remains model-limited; evidence for atherosclerosis and hepatic steatosis is functional but context/model-limited; and myocardial, renal, and other organ evidence remains largely associative or limited. Green arrows indicate functional evidence, orange dashed arrows indicate functional evidence that remains context/model-limited, and gray dotted arrows indicate associative/limited evidence. Extracellular vesicles are shown as candidate systemic carriers because their gut or microbial origin remains unconfirmed.

## 4 Clinical translation and future research directions

Most existing clinical studies are cross-sectional, making it difficult to determine whether microbiome abnormalities are a consequence of OSA and its associated hypoxic exposure, a mediator of target-organ injury, or a correlate of shared factors such as obesity and lifestyle. Future studies should therefore establish longitudinal cohorts of untreated patients with OSA and simultaneously assess hypoxic exposure, microbial function, circulating metabolites, and target-organ outcomes at baseline and during follow-up. In addition to the apnea–hypopnea index (AHI), greater emphasis should be placed on time spent with oxygen saturation below 90% (T90), the oxygen desaturation index, and hypoxic burden. Integration of metagenomic profiling with targeted metabolomics would allow temporal relationships and potential mediating pathways to be examined between microbial functional changes and cardiovascular, metabolic, neurocognitive, and renal outcomes. Existing studies suggest that hypoxemia-related indices, as well as sleep duration, sleep quality, and night-to-night variability, may be associated with microbial features (Zhang et al., 2022; Holzhausen et al., 2024; Kado, 2024). Repeated measurements are therefore likely to be more informative than single-time-point microbiome comparisons for determining the clinical relevance of microbiome alterations.

Continuous positive airway pressure (CPAP) therapy provides an important opportunity to test the reversibility of microbiome alterations, although current findings remain inconsistent. Some studies have found little evidence of substantial recovery in microbial diversity or dysbiosis-related features after CPAP treatment, whereas metagenomic studies have reported changes in microbial composition and metabolic pathways following CPAP therapy in patients with severe OSA (Bock et al., 2024; Xue et al., 2024). Future studies should objectively quantify CPAP adherence and residual hypoxemia and assess parallel changes in the microbiome, circulating metabolites, blood pressure, insulin sensitivity, hepatic steatosis, and cognitive function before and after treatment. If target-organ outcomes improve without corresponding changes in relevant microbial functions or metabolites, this would weaken, although not exclude, the hypothesis that microbiota alterations mediate the therapeutic benefit of CPAP. Conversely, if microbiome changes precede and predict improvement in organ outcomes, this would strengthen support for a functional role but would still require formal mediation analyses and targeted intervention studies.

Clinical studies must also adequately account for host, behavioral, and technical determinants of microbiome variation. Diet, obesity, metabolic status, smoking, and medication use can all substantially influence the gut microbiota (David et al., 2014; Zhernakova et al., 2016). OSA studies should therefore systematically document body-fat distribution, dietary intake and meal timing, physical activity, recent antibiotic exposure, and use of metabolically active medications, while also assessing sleep fragmentation and circadian disruption. Because hypoxia and hypercapnia may exert distinct effects on both the microbiota and organ phenotypes, carbon dioxide levels should also be monitored where feasible to distinguish isolated IH from combined intermittent hypoxia and hypercapnia (IHC) (Xue et al., 2021). In addition, sample collection, storage, sequencing, and analytical procedures should be standardized and aligned with microbiome research frameworks such as STORMS and STREAMS (Mirzayi et al., 2021; Kelliher et al., 2025) to improve reproducibility across studies.

With respect to intervention, the field should gradually move beyond empirical use of broad-spectrum probiotics toward precision microbiome strategies with defined functions and testable mechanisms. Potential approaches include specific strains or defined microbial consortia, microbial metabolites and their biosynthetic pathways, and corresponding host receptors. Candidate strategies may include restoring acetate or butyrate production, modulating bile acid transformation, inhibiting microbial trimethylamine generation, or targeting FXR, TGR5, and SCFA receptors. Baseline metagenomic and metabolomic profiles should also be incorporated to identify patient subgroups most likely to benefit rather than applying a uniform intervention to all individuals with OSA. Studies in other disease settings have demonstrated that defined microbial communities can achieve traceable intestinal engraftment, and multi-strain live biotherapeutic products have already entered early-phase clinical investigation (Dsouza et al., 2022; Silber et al., 2026). In OSA, however, their safety, engraftment stability, and effects on target-organ outcomes require dedicated clinical evaluation. Such approaches should be considered adjuncts to CPAP, weight reduction, and lifestyle modification rather than substitutes for established OSA therapies.

Overall, future research should move beyond asking whether patients with OSA exhibit differences in gut microbial composition and instead address four clinically relevant questions: which hypoxic phenotypes produce reproducible changes in microbial function; whether these changes predict target-organ injury; whether they are reversible with effective OSA treatment; and whether targeting specific strains, metabolites, or host receptors can provide independent clinical benefit. Only through a continuum of evidence spanning longitudinal association, treatment reversibility, and targeted intervention will it be possible to define the biomarker value and therapeutic potential of the gut microbiota in OSA.

## 5 Conclusion

Current evidence indicates that IH can not only directly injure target organs through tissue hypoxia, oxidative stress, and sympathetic activation, but also disrupt the intestinal oxygen environment, barrier integrity, and microbial metabolism, thereby establishing regulatory networks capable of influencing distant tissues. Compared with taxonomic alterations, which show limited consistency across studies, microbial function, metabolic output, and the associated host signaling pathways may provide a more informative framework for understanding the multisystem consequences of OSA.

However, the contribution of the gut microbiota varies substantially across target organs and is influenced by factors such as exposure pattern, diet, obesity, and sleep fragmentation. Hypertension is supported by convergent preclinical functional evidence, whereas vascular dysfunction and insulin resistance are supported by preclinical functional evidence. Neurocognitive injury has yielded mechanistically supported preclinical evidence at the levels of individual bacterial strains, microbial metabolites, and downstream immune or signaling pathways, although model-dependent limitations remain. Hepatic steatosis has preclinical functional evidence that remains context/model-limited, whereas microbiota-specific evidence for myocardial, renal, and other organ injuries is largely associative or limited. Human clinical evidence remains predominantly observational and does not yet establish microbial mediation of OSA-related target-organ injury. The gut microbiota is therefore more appropriately viewed as a context-dependent modulator and amplifier of direct IH-induced injury rather than as a uniform and indispensable mediator across all target organs. Future studies integrating longitudinal clinical cohorts, assessment of reversibility following effective OSA treatment, and interventions targeting defined microbial communities, metabolites, and host receptor pathways will be essential to determine the actual mediating contribution of the gut microbiota and its potential for precision therapeutic targeting.
