# Supplementary material for: Intermittent hypoxia–driven gut microbial and metabolic remodeling in obstructive sleep apnea: mechanisms and evidence across target organs
Source: Front Cell Infect Microbiol. 2026 Oct 26;16:1965973. doi: 10.3389/fcimb.2026.1965973 (PMC13746192; doi:10.3389/fcimb.2026.1965973)
Supplement: Supplementary file 1 [file Table1.docx]

**Supplementary Methods S1. Literature Search and Study Selection**

A structured PubMed search was conducted to identify studies relevant to this narrative review, with the final search completed in August 2026. No publication-date, language, or species restrictions were applied. MeSH and title/abstract terms related to obstructive sleep apnea and its major experimental components were combined with terms related to the gut microbiota, intestinal barrier function, and microbial or host–microbial metabolism.

**The following search strategy was used:**

Exposure-related terms

("Sleep Apnea, Obstructive"[MeSH Terms] OR "obstructive sleep apnea"[Title/Abstract] OR "obstructive sleep apnoea"[Title/Abstract] OR "obstructive sleep apnea-hypopnea syndrome"[Title/Abstract] OR "obstructive sleep apnoea-hypopnoea syndrome"[Title/Abstract] OR OSA[Title/Abstract] OR OSAHS[Title/Abstract] OR "sleep-disordered breathing"[Title/Abstract] OR "intermittent hypoxia"[Title/Abstract] OR "chronic intermittent hypoxia"[Title/Abstract] OR "intermittent hypoxemia"[Title/Abstract] OR "intermittent hypoxaemia"[Title/Abstract] OR "cyclic hypoxia"[Title/Abstract] OR "intermittent hypoxia and hypercapnia"[Title/Abstract] OR "intermittent hypoxia-hypercapnia"[Title/Abstract] OR "sleep fragmentation"[Title/Abstract])

**AND**

**Gut microbiota, barrier, and metabolic terms**

("Gastrointestinal Microbiome"[MeSH Terms] OR "gut microbiota"[Title/Abstract] OR "gut microbiome"[Title/Abstract] OR "intestinal microbiota"[Title/Abstract] OR "intestinal microbiome"[Title/Abstract] OR "fecal microbiota"[Title/Abstract] OR "faecal microbiota"[Title/Abstract] OR "fecal microbiome"[Title/Abstract] OR "faecal microbiome"[Title/Abstract] OR "gut flora"[Title/Abstract] OR dysbiosis[Title/Abstract] OR "Intestinal Barrier Function"[MeSH Terms] OR "intestinal barrier"[Title/Abstract] OR "intestinal permeability"[Title/Abstract] OR "gut permeability"[Title/Abstract] OR "Fatty Acids, Volatile"[MeSH Terms] OR "short-chain fatty acid"[Title/Abstract] OR "short-chain fatty acids"[Title/Abstract] OR SCFA[Title/Abstract] OR SCFAs[Title/Abstract] OR acetate[Title/Abstract] OR butyrate[Title/Abstract] OR propionate[Title/Abstract] OR "Bile Acids and Salts"[MeSH Terms] OR "bile acid"[Title/Abstract] OR "bile acids"[Title/Abstract] OR trimethylamine[Title/Abstract] OR "trimethylamine N-oxide"[Title/Abstract] OR TMAO[Title/Abstract] OR endotoxin[Title/Abstract] OR endotoxemia[Title/Abstract] OR endotoxaemia[Title/Abstract] OR lipopolysaccharide[Title/Abstract] OR lipopolysaccharides[Title/Abstract] OR "microbial metabolite"[Title/Abstract] OR "microbial metabolites"[Title/Abstract] OR "microbial co-metabolite"[Title/Abstract] OR "microbial co-metabolites"[Title/Abstract] OR metabolome[Title/Abstract] OR metabolomics[Title/Abstract] OR tryptophan[Title/Abstract] OR indole[Title/Abstract] OR indoles[Title/Abstract])

Records were screened by title and abstract for relevance to intestinal remodeling, microbial metabolism, and target-organ outcomes. Original human and experimental studies were considered when they examined OSA or relevant experimental exposures together with gut microbial, barrier, or metabolic measures and organ-related outcomes. Studies restricted to oral or airway microbiota or unrelated exposures were excluded; reviews and broader physiological studies were used for background, and preprints were not included in the main evidence assessment.

No target-organ terms were included in the primary search. Studies were assigned to organ sections according to the outcomes actually measured, with multiple assignments permitted when appropriate. Human and preclinical evidence and different exposure paradigms, including IH, IHC, mechanical OSA, sleep fragmentation, and dietary co-exposures, were considered separately. Both supportive and null findings were considered, with greater mechanistic weight given to transplantation, microbiota depletion or germ-free models, defined microbial or metabolite interventions, and pathway-specific perturbation. The synthesis was qualitative and narrative; no formal risk-of-bias scoring or quantitative pooling was performed.
